# Diffuse Caroli’s disease with hepatolithiasis: a case report

**DOI:** 10.1093/jscr/rjag769

**Published:** 2026-09-07

**Authors:** Shruti Sah, Ranjish Parshaila, Dinesh Nalbo, Narendra Pandit

**Affiliations:** Department of Gastrointestinal Surgery, Birat Medical College Teaching Hospital, Tankisinuwari, Morang, Biratnagar 56613, Province 1, Nepal; Department of Gastrointestinal Surgery, Birat Medical College Teaching Hospital, Tankisinuwari, Morang, Biratnagar 56613, Province 1, Nepal; Department of Gastrointestinal Surgery, Birat Medical College Teaching Hospital, Tankisinuwari, Morang, Biratnagar 56613, Province 1, Nepal; Department of Gastrointestinal Surgery, Birat Medical College Teaching Hospital, Tankisinuwari, Morang, Biratnagar 56613, Province 1, Nepal

**Keywords:** case-report, Caroli’s disease, hepatolithiasis, hepatectomy, intrahepatic biliary dilatation, Roux-en-Y hepaticojejunostomy

## Abstract

Caroli disease is characterized by segmental, non-obstructive dilatation of the intrahepatic bile ducts, leading to bile stasis, recurrent cholangitis, hepatolithiasis, and an increased risk of cholangiocarcinoma. Caroli’s disease, together with hepatic fibrosis, is termed Caroli’s syndrome, which may lead to portal hypertension. Caroli’s disease often requires treatment in symptomatic patients. Symptoms include recurrent cholangitis due to intrahepatic stones in dilated ducts or cirrhosis due to fibrosis. Liver transplantation remains the definitive treatment for diffuse disease. However, owing to organ shortages and long waiting lists, liver resection is an alternative for localized disease. Here, we present a rare case of an 18-year-old male with symptomatic, right-lobe-predominant Caroli’s disease and hepatolithiasis who underwent surgical resection (right hepatectomy, stone extraction from exposed ducts at the resection margin, and intrahepatic-jejunostomy) with symptom resolution. This case highlights the role of liver resection in localized disease and reinforces the importance of individualized interventions in resource-limited settings.

## Introduction

Caroli disease is a rare, congenital condition characterized by segmental dilatation of large, intrahepatic bile ducts, which appear as cysts on imaging studies and histopathological examination [1, 2]. This condition is part of the fibropolycystic liver diseases spectrum, resulting from ductal plate malformations during embryonic development. It can present in two forms: the simple form, involving only bile duct dilatation, and Caroli syndrome, which combines bile duct dilatation with congenital hepatic fibrosis [3, 4]. Diagnosis typically involves imaging modalities like ultrasonography, computed tomography (CT), and magnetic resonance cholangiopancreatography (MRCP), which reveal the characteristic bile duct abnormalities [5]. The entity can present with symptoms of abdominal pain, recurrent cholangitis of liver failure due to end-stage liver disease. The best treatment for the entity is liver transplantation in advanced cases, but liver resection remains an option for localized disease. Here we present a rare case of a young adult presenting with recurrent pain in the abdomen without cirrhosis, managed successfully with liver resection.

## Case presentation

An 18-year-old male presented with recurrent episodes of dull-aching right hypochondrial pain over 3 years. The pain was non-radiating, gradually progressive, and not associated with jaundice, nausea, or vomiting. The patient also reported intermittent fever without chills or rigors. On general physical examination, he was hemodynamically stable and afebrile, with no evidence of jaundice, pallor, clubbing, cyanosis, or edema. Baseline hematological and biochemical investigations, including liver function tests, were within normal limits.

**Figure 1:**
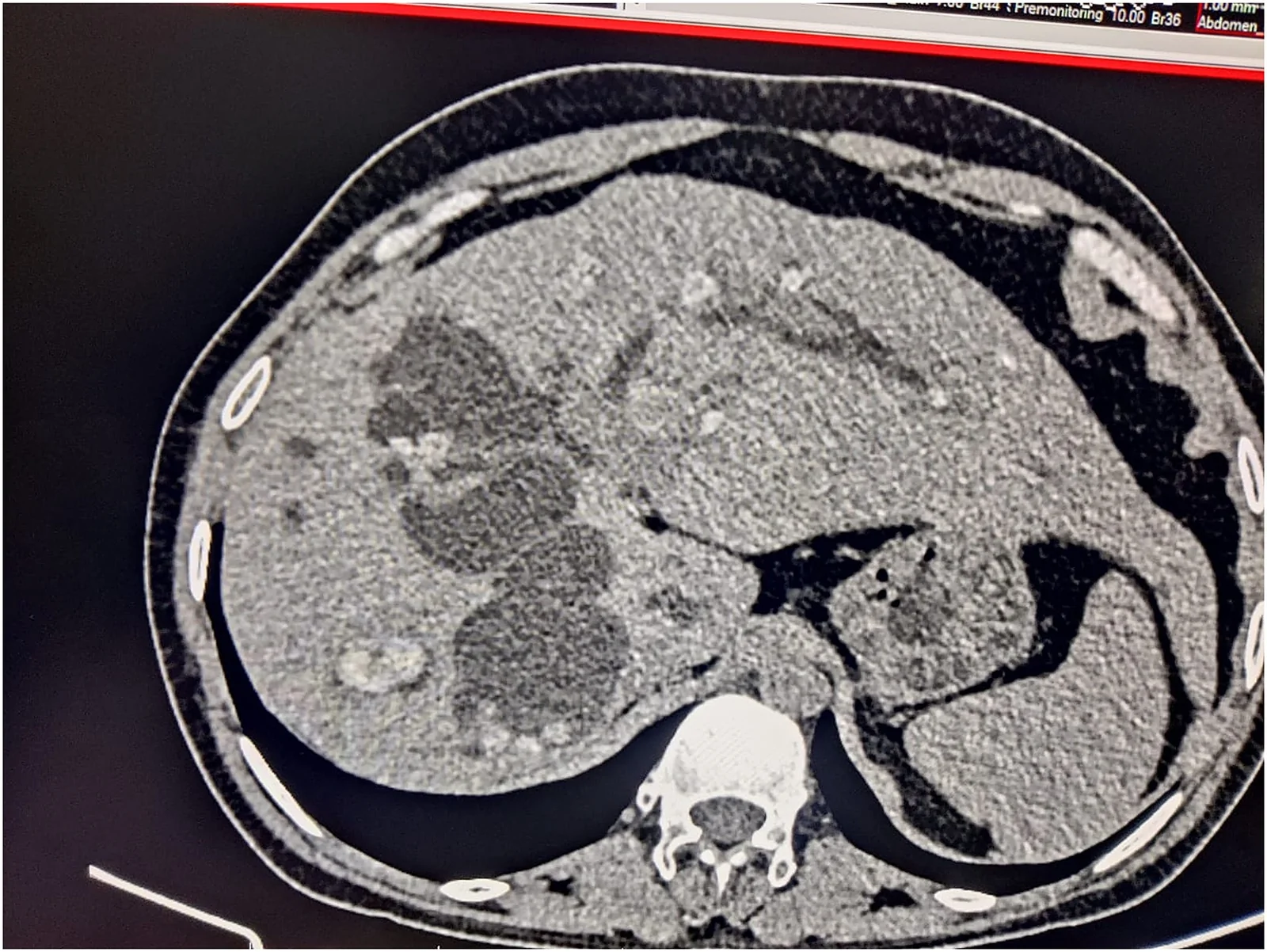
CECT image showing intrahepatic biliary dilatation (right predominant) with multiple hepatolithiasis.

**Figure 2:**
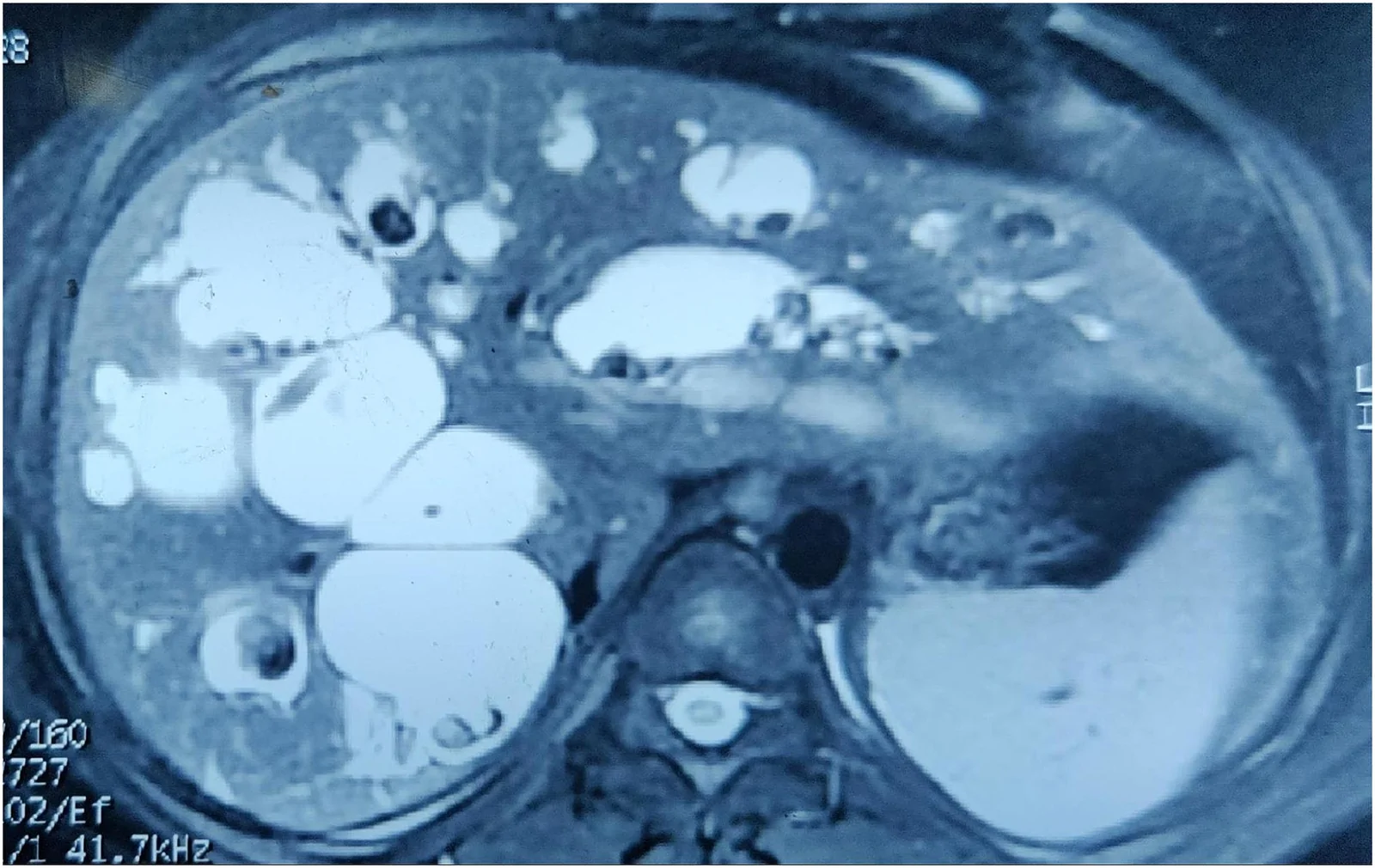
MRCP image showing right predominant disease with grossly dilated intrahepatic ducts with stones.

**Figure 3:**
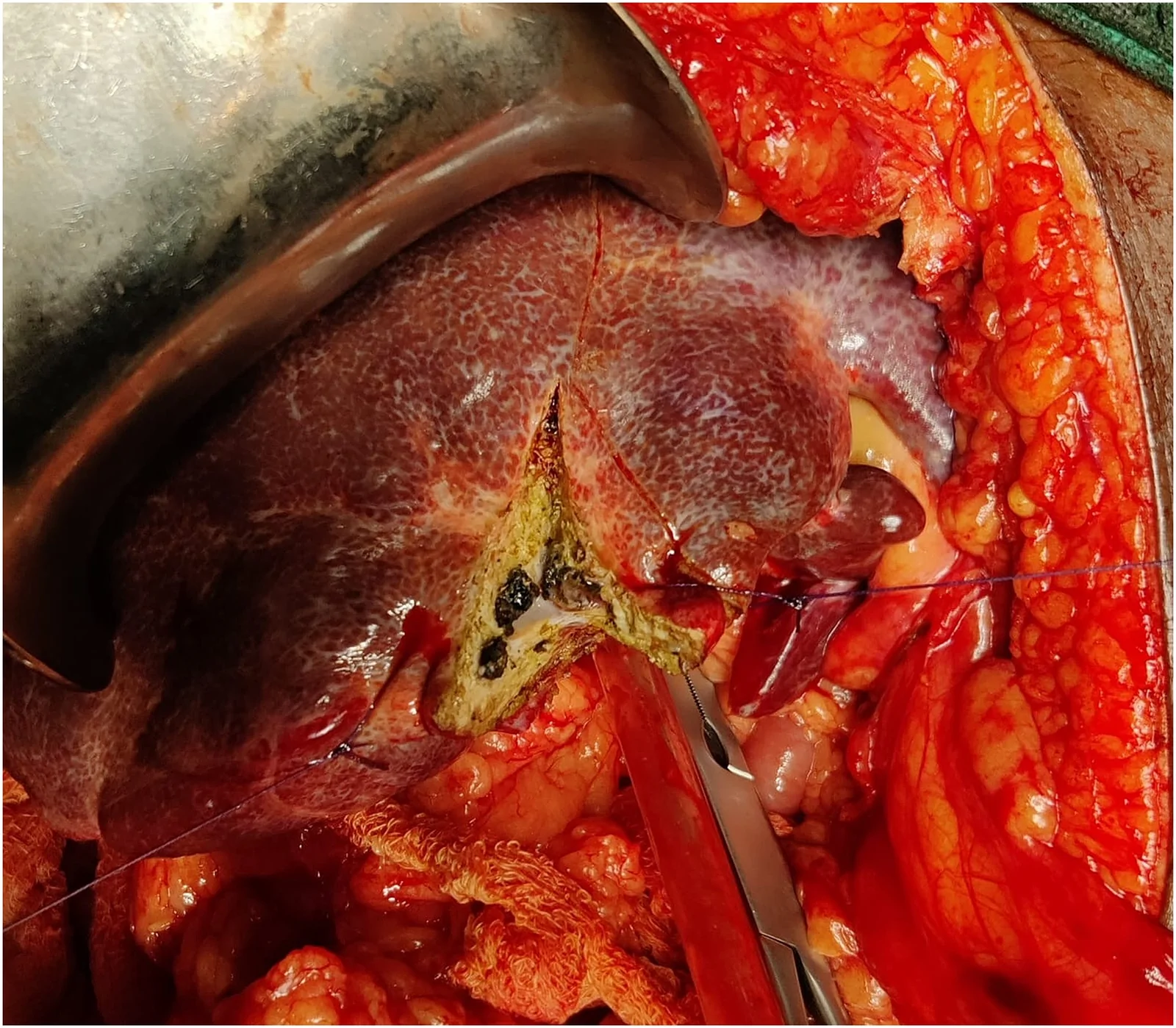
Image showing dilated intrahepatic ducts with pigment stones at the transection margin.

**Figure 4:**
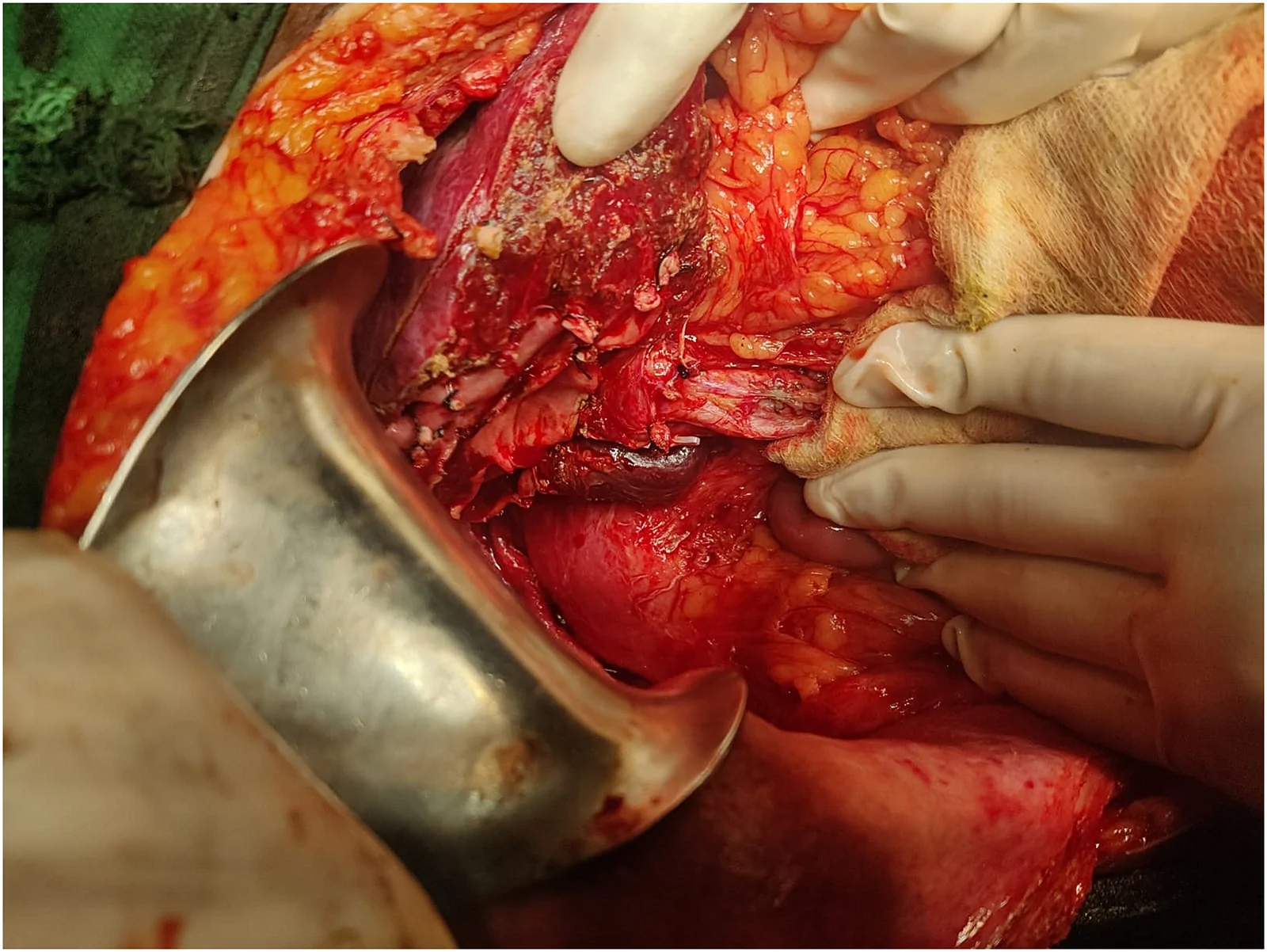
Post resection view demonstrating dilated ducts in the liver remnant following right hepatectomy.

**Figure 5:**
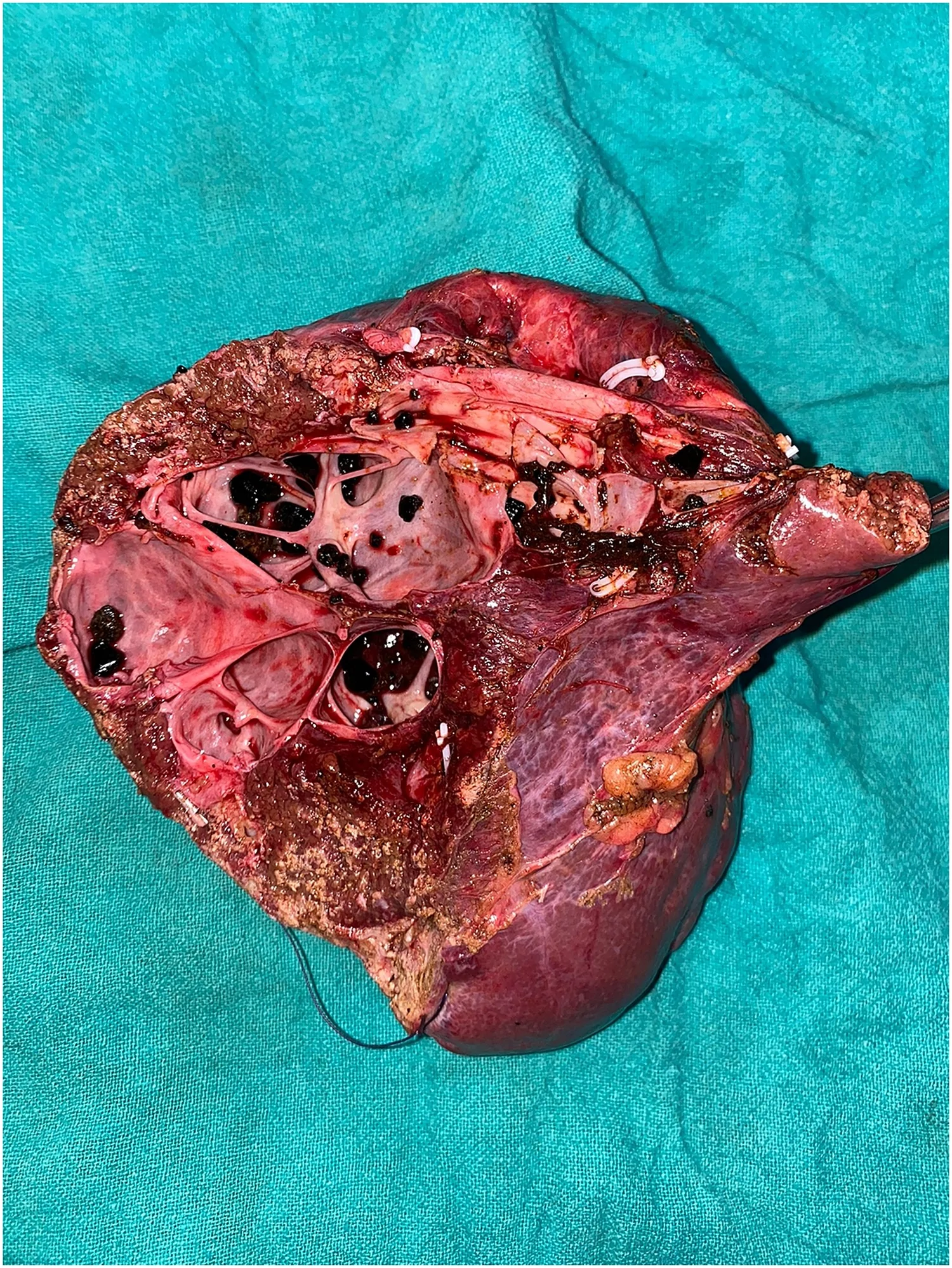
Resected right liver with stones within the dilated ducts.

Abdominal ultrasonography revealed dilated intrahepatic biliary radicals without choledocholithiasis. To further delineate the biliary anatomy and pathology, contrast-enhanced computed tomography (CECT) of the abdomen was performed, which demonstrated dilated intrahepatic bile ducts with multiple stones (Fig. 1). The findings were further confirmed with MRCP (Fig. 2). The endoscopy excluded features of portal hypertension (esophageal varices). Considering the persistent symptoms of abdominal pain and young age, some intervention needed to be considered. As the disease was localized to the right liver, and packed with intrahepatic stones, we planned for right hepatectomy. The patient although was a candidate for liver transplantation, they denied due to the financial constraints. The surgery was performed with a right Makuuchi incision. Intraoperatively, the liver was normal, right hepatectomy was done; multiple dilated ducts were exposed along the transection plane with packed stones (Fig. 3), which were removed. Resected liver had multiple pigmented stones inside the dilated ducts. At the dependent part of the right liver, Roux-en-Y intrahepatic jejunostomy was done to the exposed part of the dilated bile ducts (Figs 4 and 5). Operating time was 4 h and blood loss of 350 ml. Postoperatively, the patient had low-volume bile leak, which subsided over 14 days with conservative management. Histopathology of the resected liver confirmed recurrent pyogenic cholangitis, without any features of malignancy. Further, there was no evidence of liver fibrosis. After 3 years of follow-up, he is doing good. The patient remained asymptomatic with no recurrence of symptoms or complications.

## Discussion

The halted development of large bile ducts leads to the formation of intrahepatic cysts. These abnormally developed ducts become significantly enlarged, resulting in bile stasis. This stagnation promotes the accumulation of biliary sludge and the formation of hepatoliths, increasing the risk of infections and repeated episodes of cholangitis [6, 7].

Though rare, the incidence of Caroli disease is estimated to be 1 in 1 000 000 live births and is more prevalent in regions where hepatolithiasis is endemic [8, 9].

Although medical therapy remains the mainstay for asymptomatic or mildly symptomatic patients, surgery is considered in localized disease with recurrent cholangitis or stone formation. Hepatic resection is curative in selected cases and prevents complications such as secondary biliary cirrhosis and cholangiocarcinoma. The risk of malignancy in patients with Caroli disease is estimated to range between 7% and 14%, particularly in long-standing or diffuse disease [10, 11]. Additionally, secondary biliary cirrhosis is a significant concern due to chronic inflammation and biliary obstruction. Though precise rates vary, studies suggest that cirrhosis may develop in untreated or advanced cases.

Radiological evaluation using ultrasound, CT, and MRCP plays a crucial role in diagnosis. The presence of cystic intrahepatic biliary dilatations and the characteristic ‘central dot sign,’ which reflects the fibrovascular bundles within the dilated ducts, are considered diagnostic.

Treatment approaches should be tailored based on the extent of liver involvement and the feasibility of surgical options. Medical modality includes antibiotics for cholangitis, ursodeoxycholic acid for bile flow, and endoscopy for stone extraction. Surgical modalities include liver resection which is curative in localized disease, and liver transplantation which is indicated in diffuse, bilateral disease or in the presence of cirrhosis/cholangiocarcinoma [12–16].

In our case, despite the bilateral disease process, the pathology was present predominantly to the right liver, which allowed for right hepatectomy with the resolution of symptoms and an excellent long-term outcome. Further, we added intrahepatic jejunostomy to drain the dilated ducts which has rarely been reported in literature [17]. This case underscores the importance of early recognition and surgical management of focal Caroli disease, especially in young patients where curative resection can significantly improve quality of life and prevent long-term complications.

## Conclusion

Caroli’s disease is a rare entity and defined as type V choledochal cyst in Todani’s classification. They may present with non-specific abdominal pain due to hepatolithiasis. Liver resection is the treatment for symptomatic patients with predominant unilateral disease. Intrahepatic jejunostomy to remnant liver further prevents recurrence of intrahepatic stones. This case also highlights the importance of intraoperative assessment and decision-making regarding biliary reconstruction, with intrahepatic Roux-en-Y hepaticojejunostomy offering a long-term drainage route and minimizing recurrence risk.

## References

[1] Lefere M, Thijs M, De Hertogh G et al. Caroli disease: review of eight cases with emphasis on magnetic resonance imaging features. Eur J Gastroenterol Hepatol 2011;23:578–85. 10.1097/MEG.0b013e3283470fcd 21543986

[2] Kyalwazi B, Kudaravalli P, John S. Caroli Disease. In: StatPearls [Internet]. Treasure Island (FL): StatPearls Publishing, 2025. Available from: https://www.ncbi.nlm.nih.gov/books/NBK513307/. 30020679

[3] Agustsson AI, Cariglia N. Sjúkdómur Carolis--sjúkratilfelli og yfirlit fraedigreina [Caroli's disease, case report and review of the literature]. Laeknabladid 2007;93:603–5. Icelandic. 17823500

[4] Yilmaz S, Kirimlioglu H, Kirimlioglu V et al. Partial hepatectomy is curative for the localized type of Caroli's disease: a case report and review of the literature. Surgeon 2006;4:101–5. 10.1016/s1479-666x(06)80039-9 16623167

[5] Shafqat MN, Memon MYY, Javed S et al. Caroli's syndrome: a case report and literature review. Cureus 2023;15:e50871. 10.7759/cureus.50871 38249206 PMC10799222

[6] Brancatelli G, Federle MP, Vilgrain V et al. Fibropolycystic liver disease: CT and MR imaging findings. Radiographics 2005;25:659–70. 10.1148/rg.253045114 15888616

[7] Desmet VJ . Congenital diseases of intrahepatic bile ducts: variations on the theme “ductal plate malformation”. Hepatology 1992;16:1069–83. 10.1002/hep.1840160434 1398487

[8] Khan MZ, Kichloo A, El-Amir Z et al. Caroli disease: a presentation of acute pancreatitis and cholangitis. Cureus 2020;12:e9135. 10.7759/cureus.9135 32789075 PMC7417087

[9] Cabral Correia P, Morgado B. Caroli's disease as a cause of chronic epigastric abdominal pain: two case reports and a brief review of the literature. Cureus 2017;9:e1701. 10.7759/cureus.1701 29159008 PMC5690396

[10] Millwala F, Segev DL, Thuluvath PJ. Caroli's disease and outcomes after liver transplantation. Liver Transpl 2008;14:11–7. 10.1002/lt.21366 18161799

[11] Phinney PR, Austin GE, Kadell BM. Cholangiocarcinoma arising in Caroli's disease. Arch Pathol Lab Med 1981;105:194–7. 6260057

[12] Nagasue N . Successful treatment of Caroli's disease by hepatic resection. Rep Six Patients Ann Surg 1984;200:718–23. 10.1097/00000658-198412000-00008 PMC1250588 6508401

[13] Kassahun WT, Kahn T, Wittekind C et al. Caroli's disease: liver resection and liver transplantation. Experience in 33 patients. Surgery 2005;138:888–98. 10.1016/j.surg.2005.05.002 16291390

[14] Zahmatkeshan M, Bahador A, Geramizade B et al. Liver transplantation for caroli disease. Int J Organ Transplant Med 2012;3:189–91. 25013645 PMC4089297

[15] Yonem O, Bayraktar Y. Clinical characteristics of Caroli's syndrome. World J Gastroenterol 2007;13:1934–7. 10.3748/wjg.v13.i13.1934 17461493 PMC4146969

[16] Fahrner R, Dennler SG, Inderbitzin D. Risk of malignancy in Caroli disease and syndrome: a systematic review. World J Gastroenterol 2020;26:4718–28. 10.3748/wjg.v26.i31.4718 32884228 PMC7445861

[17] Nagino M, Nishio H, Ebata T et al. Intrahepatic cholangiojejunostomy following hepatobiliary resection. Br J Surg 2007;94:70–7. 10.1002/bjs.5531 17058317

